# Water surface garbage detection based on lightweight YOLOv5

**DOI:** 10.1038/s41598-024-55051-3

**Published:** 2024-03-13

**Authors:** Luya Chen, Jianping Zhu

**Affiliations:** 1 College of Engineering Science and Technology, Shanghai Ocean University, Shanghai, 21306 China; 2 School of Engineering, Shanghai Ocean University, Shanghai, 21306 China

**Keywords:** Garbage detection, Lightweight model, Attention mechanism, Lightweight convolution, Ecology, Engineering

## Abstract

With the development of deep learning technology, researchers are increasingly paying attention to how to efficiently salvage surface garbage. Since the 1980s, the development of plastic products and economic growth has led to the accumulation of a large amount of garbage in rivers. Due to the large amount of garbage and the high risk of surface operations, the efficiency of manual garbage retrieval will be greatly reduced. Among existing methods, using YOLO algorithm to detect target objects is the most popular. Compared to traditional detection algorithms, YOLO algorithm not only has higher accuracy, but also is more lightweight. This article presents a lightweight YOLOv5 water surface garbage detection algorithm suitable for deployment on unmanned ships. This article has been validated on the Orca dataset, experimental results showed that the detection speed of the improved YOLOv5 increased by 4.3%, mAP value reached 84.9%, precision reached 88.7%, the parameter quantity only accounts for 12% of the original data. Compared with the original algorithm, the improved algorithm not only has higher accuracy, but also can be applied to more hardware devices due to its lighter weight.

## Introduction

According to a research survey^1^, China is one of the countries with the highest number of rivers in the world, with a total river area of approximately 5.098 million square kilometers, accounting for 53.1% of the country's total area. With rapid economic development and the acceleration of urbanization, the problem of water surface garbage pollution has become increasingly severe, posing a serious threat to water environments, transportation, and urban landscapes. The hazards of water surface garbage can be summarized as follows. Firstly, when water surface garbage remains immersed in water for a long time, it can release harmful substances and affect water quality. Secondly, a large amount of floating garbage covering the water surface can block sunlight from entering the water, thereby impacting the ecological balance. Thirdly, the presence of a significant amount of water surface garbage, especially white garbage, severely damages the aesthetic appeal of the water surface, thereby affecting the development of local tourism. Lastly, floating debris in waterways can obstruct vessel navigation, and entanglement with propellers can jeopardize vessel safety. Nowadays, pollution control of inland water garbage has become one of the key concerns for governments at all levels, and the urgent need to clean and dispose of water surface garbage cannot be delayed.

Based on this, this paper proposes a water surface garbage detection algorithm suitable for deployment on unmanned boats to address the issue of insufficient computing resources on unmanned boats and achieve automatic detection of water surface garbage. The lightweight YOLO algorithm not only enables real-time and accurate detection, but also frees human resources from dangerous and arduous tasks. What's more, due to the lightweight of the improved algorithm, it requires lower hardware resource demands, making it adaptable to different types of unmanned boats.

The use of object detection algorithms on unmanned ships has been proposed by this paper, with the aim of reducing waste of human resources and reducing the risk of surface operations. Target detection algorithms mainly include methods based on candidate regions and non-candidate regions. Methods based on candidate regions typically use a two-step detection method. Firstly, a series of bounding boxes are generated from the input image, and then convolutional neural networks are used to extract features from the generated region and construct an object classifier. Finally, classify and regress the candidate regions. Its main representative detection algorithms include Faster RCNN^2^ and Mask RCNN^3^. In 2016, Ren et al.^2^ proposed the Fast RCNN detection algorithm, which has higher detection accuracy, especially for small object detection, and has higher detection performance. However, the detection algorithm uses a fully connected network, which leads to an increase in computation parameters and thus increases hardware requirements. In 2017, He et al.^3^ proposed the Mask RCNN algorithm, which introduces the mask branch on the basis of Faster RCNN and can perform instance segmentation while performing object detection. However, this algorithm requires a large amount of computing resources, which leads to high hardware requirements. Moreover, Mask RCNN has poor real-time performance due to the need for a large amount of computation. The method based on noncandidate regions treats the target detection method as a regression problem, and uses convolutional neural networks to directly predict the probability of bounding boxes and classes from the complete image. The main representative algorithms include SSD and YOLO series algorithms. In 2016, Liu et al.^4^ proposed the SSD algorithm. Compared to candidate region based object detection algorithms, SSD algorithm has a faster detection speed, but its detection performance is poor for small targets and targets with high overlap. The YOLO algorithm has been iterated to the eighth generation YOLOv8, which uses a separate CNN model to achieve end-to-end detection. This algorithm has high detection speed and accuracy. In addition, compared to the Faster RCNN algorithm, the YOLO series algorithm is lighter and computes fewer parameters. After data analysis and comparison, and considering the issue of insufficient hardware resources for unmanned ships, YOLOv5 algorithm has more advantages, the YOLOv5 algorithm in the YOLO series is more suitable for deployment on unmanned ships.

To make the model lighter, a lightweight network is generally used instead of the original network structure of YOLOv5. The currently mature lightweight network structures include: Shufflenet, Mobilenet, Efficientnet, and Ghostnet. The Shuflenetv1 network structure was first proposed by the Zhang^5^ team in 2017. On this basis, the Zhang^6^ team proposed the Shufflenetv2 network architecture in 2018. Shufflenetv2 separates convolutions through the DWConv module, achieving lightweight network performance; through channel mixing operation, information exchange is achieved to improve the receptive field. Andrew Howard^7^ team proposed the Mobilenetv3 Large and Mobilenetv3 Small lightweight network structures in 2019, which were designed for high and low resource usage, respectively. For semantic segmentation tasks, Mobilenetv3 proposes a new effective segmentation decoder Lite Reduced Atrus Spatial Pyramid Pooling (LR-ASPP). Compared with Mobilenetv2^8^, Mobilenetv3 Large and Mobilenetv3 Small have improved accuracy in image classification and object detection, and significantly reduced latency. The Efficientnet lightweight network structure was first proposed by Tan et al.^9^ in 2019. Unlike traditional scaling model methods, this network structure adopts a simple and efficient composite scaling method. After verification, the Efficientnet network structure performs better on the ImageNet dataset, with higher accuracy, faster detection speed. Moreover, compared to traditional convolutional network structures, the Efficientnet network structure is more lighter. The Ghostnet network structure was first proposed by Han^10^ team in 2019. This network structure uses the Ghost module to perform a series of linear transformations, generating many ghosting feature maps to fully reveal the information behind the feature maps. On the ImageNet dataset, the Ghostnet network structure has higher accuracy and lighter weight compared to the Mobilenetv3 network structure.

Although the network structure mentioned above is lightweight, its accuracy still cannot surpass the original structure. For this situation, most scholars integrate attention mechanisms into lightweight network structures to improve detection accuracy. At present, the more mature attention mechanisms include SE (Squeeze and Excitation) attention mechanism, CA (Channel Attention) attention mechanism, and CBAM (Convolutional Block Attention Module) attention mechanism. The SE attention mechanism was proposed by Hu^11^, which focuses on the channel dimension and can adaptively adjust channel weights, making the model more focused on useful information. The CA attention mechanism was proposed by Hou^12^. This attention mechanism focuses on both channel and spatial dimensions, and can autonomously learn channel weights, making the model more focused on effective information. The CBAM attention mechanism was proposed by Woo^13^, which includes two sub modules: CAM (Channel Attention Module) and SAM (Spatial Attention Module), which focus on the channel and space of the model, respectively. This attention mechanism is a plug and play module that not only saves computational costs, but also enables the model to focus on more effective information.

With the development of deep learning, many scholars at home and abroad have combined object detection algorithms with garbage detection and applied them to daily life. Mei^14^ proposed a lightweight garbage detection network based on YOLOv5 to address the difficulties and low efficiency of manual sorting of garbage. This network achieves lightweight while ensuring accuracy. Jiang^15^ proposed an attention combination mechanism network structure based on YOLOv5 algorithm to address the issues of high complexity, serious missed detection of small targets, and low real-time performance of garbage detection models. The improved algorithm has better detection speed and accuracy, reducing the complexity of the network model to a certain extent. Jin^16^ proposed to establish a machine vision system based on improved MobileNetV2 network, in order to reduce labor costs and improve waste sorting capability. The algorithm has a high accuracy rate on the data set, and the model volume is only 30.1% of the MobileNetV2 module. Cai^17^ studied and established plastic waste detection models to realize the automatic capture of marine plastic waste. The detection model improves the ability of target feature extraction and generalization, and solves the problems of slow rate of convergence and low training efficiency. Zhao et al.^18^ proposed a Skip-YOLO model suitable for domestic waste detection, which uses large convolutional kernels to expand the receptive field of the model and enhance the shallow information of the image. Compared with YOLOv3, the accuracy is increased by 22.5% and the recall rate is increased by 18.6%. To solve the problem of insufficient manpower for urban garbage disposal, Vivekanandan et al.^19^ proposed a new robot navigation technology. The author proposes to use moving edge computing to process the street image in advance, filter out the target image, use SSD to segment the ground, and finally use HOG to extract features. The experimental results show that using robots for navigation and garbage detection is more efficient than traditional methods. To solve the problem of difficult fishing for marine plastic waste, Damayanti et al.^20^ proposed combining the Kernel Normalized Difference Vegetation Index (KNDVI) and Floating Fragment Index (FDI) in satellite images, and then using Sentinel-2 multispectral instrument (MSI) to detect Marine plastic waste. The results indicate that the combination of KNDVI and FDI can effectively detect marine plastic waste on the sea surface. Fayaz^21^ proposed using machine learning for spam classification. The machine learning model proposed by Muhammad Fayaz integrates multi-layer perceptron (MLP), K-nearest neighbor, and random forest (RF) algorithms. The experimental results show that the classification accuracy of the integrated model proposed by Fayaz is better than that of a single model. Adnan^22^ proposed a high-performance and highly accurate machine learning algorithm for data mining. The ML algorithm proposed by Muhammad Adnan can predict student performance, which is suitable for teacher teaching and provides customized guidance for students. According to surveys, sonar is often used for marine debris detection tasks and underwater scene analysis. However, achieving fully supervised denoising and garbage detection in sonar detection is still a challenging task. Keyang^23^ proposed a sonar garbage detection model that combines global speckle removal and dynamic attention optimization. The experimental results on the ARACATI2017 and marine debris datasets show that the algorithm model proposed by Keyang has improved noise reduction by 0.428 and garbage detection accuracy by 4.2%.

Traditional garbage fishing requires a lot of manpower and resources, and is not very real-time and has high risks. This article proposes an unmanned ship system with autonomous detection capability, and our team will continue to research how to design unmanned ships in the future. Chunyu^24^ designed an unmanned ship system with autonomous navigation and water management capabilities. The unmanned ship system adopts an IoT framework design, combined with a measurement and control system, which can upload water quality data in real time to cloud servers, and the mobile app can display the data in real time. To solve the problem of water resource pollution, Yadong^25^ has designed a visual recognition based water surface garbage cleaning device specifically applied to the ocean. The unmanned ship designed by Zhou Yadong uses visual recognition for garbage detection and compresses garbage through a garbage compressor. To solve the problem of artificial fishing garbage not being able to enter complex river basins, Yunshuai^26^ proposed deploying surface garbage detection algorithms on unmanned ships. The detection algorithm uses the optimized YOLOv5s algorithm. Ding Yunshuai uses the CBAM module to suppress the interference of water surface reflection, and adds the DeepSort tracking algorithm to reduce the missed detection rate. In addition, introducing the EIoU loss function into the network enables the model to regress more accurately. The results show that the improved algorithm has higher accuracy and faster speed. The unmanned ship designed by Yunshuai includes a power module, a power module, a communication module, and a sensor module. The processor uses ARM A9 + Nvidia GPU. The use of unmanned boats equipped with target detection algorithms not only realizes the automation of surface garbage detection, but also uses unmanned boats to go to dangerous waters for garbage cleanup, thereby reducing the risk of water operations. To address the issue of data security not being guaranteed and preference feedback being ignored, Yu^27^ proposals secure AIoT for implicit group recommendations (SAIoT GRs). This security architecture can maximize the advantages of the Internet of Things (IOT) structure and ensure the security of data transmission. Mast^28^ found that channel contention can lead to packet loss in WANET. To address this issue, Noor Mast proposed a channel competition based routing protocol (CCBR). The results indicate that CCBR is superior to AODV in terms of packet transmission rate and end-to-end latency. To address the issue of insecure data storage, Yu^29^ proposes a Shamir threshold cryptography scheme for IIoT data protection using blockchain: STCChain. The results indicate that STCChain can effectively prevent attackers from stealing data and ensure data security. Aziz^30^ proposed global and local extensions of some commonly used local similarity indices. The results indicate that the extension proposed by Furqan Aziz has superior performance. To address the issues of low accuracy, slow processing speed, and poor generalization of phase extraction methods in Electronic Speckle Pattern Interchange (ESPI), a method that combines deep learning with phase extraction is proposed by Wenbo Jiang^31^. The advantages and disadvantages of applying deep learning technology to phase extraction algorithms have been demonstrated. To address the issue of unsafe navigation of ships in inland rivers, Yuanzhou Z^32^ studies Recognition and Depth Estimation of Ships Based on Binocular Stereo Vision. In the ship recognition stage, the algorithm uses MobileNetV1 as the backbone network of the YOLOv4 model to reduce computational complexity. In the depth estimation stage, the FSRCNN algorithm is used to perform super-resolution reconstruction on the original image, further increasing the detection accuracy. After investigation, it was found that most dual-modality object detection algorithms ignored modal differences, resulting in incomplete modal feature extraction and insufficient fusion. For this reason, R Zhang^33^ proposes a new Differential Feature Awareness Network (DFANet) for infrared and visible light object detection. Experiments have shown that DFANet has stronger robustness and superiority.

The rest of this paper is structured as follows: Section "Proposed methodology" introduces the original model and the Shufflenetv2 network structure, SE attention module, GSConv and VoV-GSCSP module used in the improved model. In Section "Results", the indicators of the model were presented and the improved model was validated. In Section "Apply", a design diagram for unmanned ships was presented, and the feasibility and challenges of deploying detection algorithms on unmanned ships were discussed. Finally, the conclusion and discussion are presented in “Conclusion”.

## Proposed methodology

### YOLOv5 model structure

YOLOv5 has five model structures: YOLOv5n, YOLOv5s, YOLOv5m, YOLOv5l, and YOLOv5x. Its network model structure is roughly the same, but its network depth and width is different. The smallest model structure is YOLOv5n, and the largest model structure is YOLOv5x.

**Figure 1:**
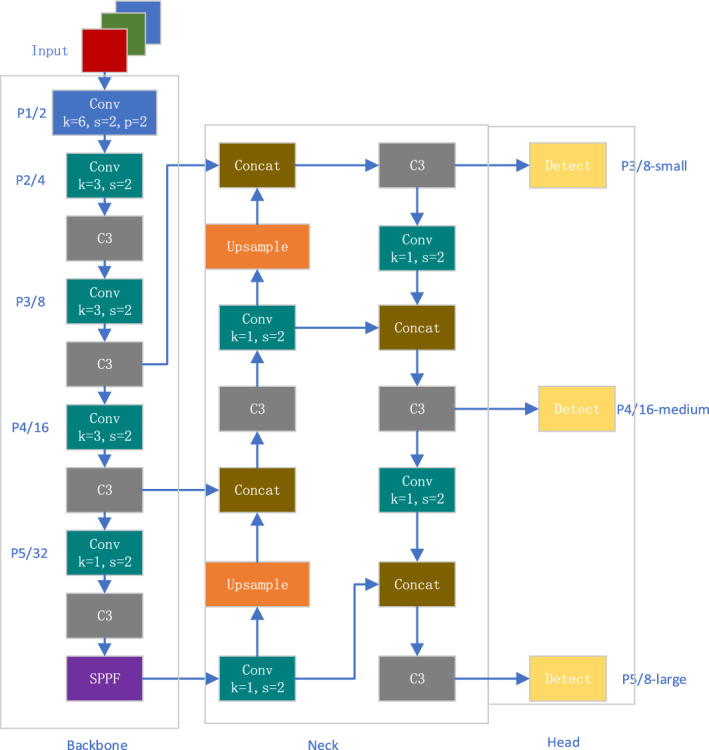
YOLOv5s model structure.

As shown in Fig. 1, the algorithm framework includes Backbone, Neck, and Head. Backone uses the CSP-Darknet53 network structure, which is mainly composed of Conv module, C3 module, and SPPF (Spatial Pyramid Pooling Module). The Conv module is a standard convolutional module which is composed of BN and SiLU activation functions. The C3 module is the main module for learning residual features. Its structure is divided into two branches. One uses multiple BottleneckCSP stacks and three standard convolutional layers, and the other only passes through one basic convolutional module. Finally, the two branches are concated. When inputting images, the network structure mainly performs downsampling operations on the feature map, extracts image features, through the residual network structure learns residual features to obtain more information. Finally, passes the feature information to the Neck section through the SPPF module.

The Neck feature pyramid adopts FPN and PAN structures. It combines the methods of FPN conveying strong semantic features from top to bottom and PAN conveying strong localization features from bottom to top, and aggregates parameters for different detection layers, improving the receptive field and enriching the expression ability of feature maps.

The Head object detection head is used to detect feature maps. The Head section uses CIOU_ LOSS as the loss function of the bounding box, and uses weighted NMS method to filter the target box, finally obtaining the appropriate target box.

#### Loss function

The YOLOv5 network model uses the CIOU loss function. CIoU considers three factors: the overlap area, aspect ratio, and center point distance between the predicted box and the real box, which improves the regression accuracy on the basis of DIoU. The calculation formula for the CIoU loss function is:

1 $$L_{{CI{\text{oU}}}} = 1 - IoU + (\frac{{\rho^{2} (b,b^{gt} )}}{{c^{2} }}) + \alpha \upsilon$$

Among them, $b,b^{gt}$ represents the center points of the prediction box and the real box, $\rho$ represents the Euclidean distance between them, c represents the diagonal distance of the smallest box containing both the prediction box and the real box, $\alpha$ is a weight parameter, $\upsilon$ is a parameter used to measure the similarity of aspect ratio, and IoU is the intersection and union ratio between the predicted box and the real box.

The calculation formula for α is:

2 $$\alpha = \frac{\upsilon }{(1 - IoU) + \upsilon }$$

The calculation formula for υ is:

3 $$\upsilon = \frac{4}{{\pi^{2} }}\left( {\arctan \frac{{w^{gt} }}{{h^{gt} }} - \arctan \frac{w}{h}} \right)^{2}$$

### Algorithm improvement

**Figure 2:**
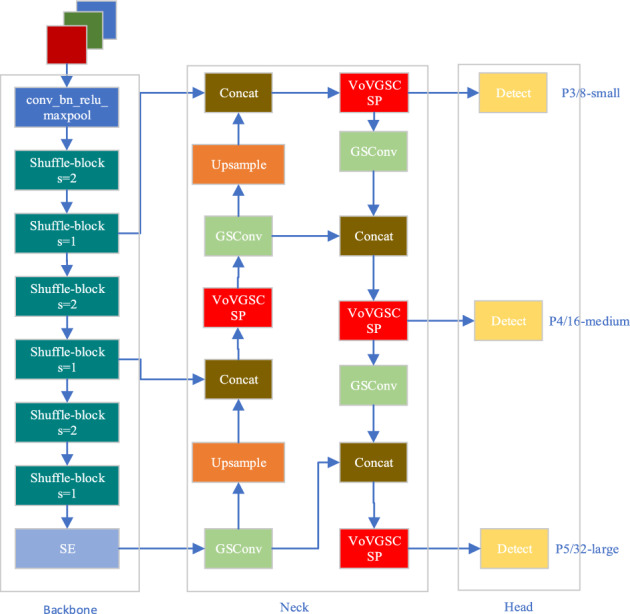
Improved YOLOv5s model structure.

This article proposes a lightweight garbage detection algorithm based on YOLOv5. The improved algorithm combined Shufflenetv2 lightweight network with SE attention mechanism as the backbone network which reduce the network model and improve the detection speed. In the Neck section, this article uses the GSConv module to replace the Conv module, and also uses the VoVGSCSP module to replace the C3 module, which not only makes the model lighter, but also improves accuracy. The improved YOLOv5s model structure is shown in Fig. 2.

#### Shufflenetv2 basic unit

In 2017, Xiangyu et al.^5^ proposed an efficient mobile device convolutional neural network-Shufflenet network structure, which is mainly applied to mobile devices with poor computing power. In 2018, Xiangyu et al.^6^ further proposed an optimized Shufflenetv2 network structure based on the Shufflenet network structure, and designed four lightweight network principles.

**Figure 3:**
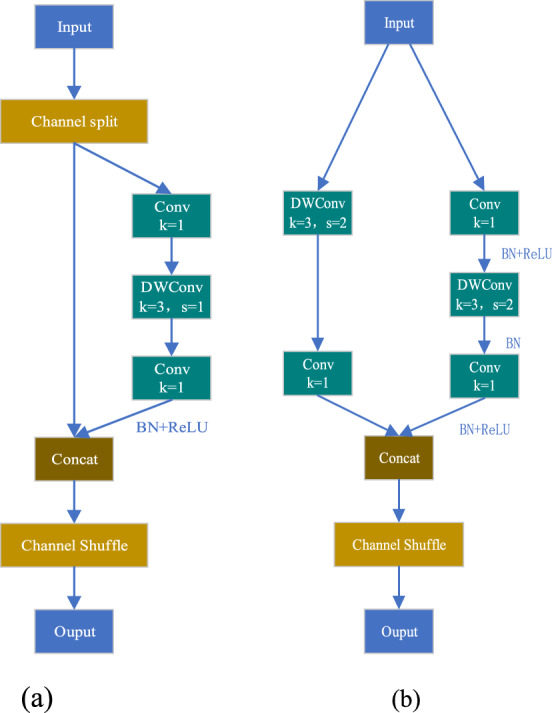
Shufflenetv2 basic module, where a represents the structure of side = 1 and b represents the structure of side = 2.

The structure of Shufflenetv2 is shown in Fig. 3. When stride = 1, first use the channel split operation to evenly divide the input channel count into two halves, with the left branch performing a constant mapping, and the right branch consisting of three convolutions. After convolution, connect two branches to ensure that the number of output channels remains unchanged. Finally, the channel information is disrupted through the channel shuffle operation to ensure the exchange of information between the two branches. When stride = 2, it is the down sampling unit of Shufflenetv2. It differs from the basic unit in that it cancels the channel split operation, doubles the number of channels.

The two key points in Shufflenetv2 are Depthwise Convolution (DWConv) and Channel Shuffle. DWConv efficiently achieves model lightweighting, while Channel Shuffle facilitates information interaction and enhances receptive field.

DWConv is a special convolutional technique. Unlike standard convolution, each channel has its own unique kernel for the convolution operation, rather than all channels sharing a single kernel. In standard convolution, assuming the input image size is $W \times H \times C$, the kernel size is $K \times K$, and the number of kernels is N, the total number of parameters can be calculated as follows:

4 $${\text{P}}_{{{\text{Conv}}}} = K \times K \times C \times N$$

DWConv groups the input feature maps and then convolves them. Assuming they are divided into x groups, the size of the input feature maps is $W \times H \times \frac{C}{{\text{x}}}$, the required number of convolution kernels is $\frac{K}{{\text{x}}}$, and the resulting parameter quantity is:

5 $$P_{{DWC{\text{onv}}}} = W \times H \times \frac{C}{{\text{x}}} \times \frac{K}{{\text{x}}} \times {\text{x}} = \frac{W \times H \times C \times K}{{\text{x}}}$$

Comparing formulas (4) and (5), it is found that the number of parameters for convolution using DWConv only accounts for $\frac{1}{{\text{x}}}$ of the standard convolution, indicating that using grouped convolution can effectively reduce the number of model parameters and make the model lighter.

#### Squeeze and excitation module

**Figure 4:**
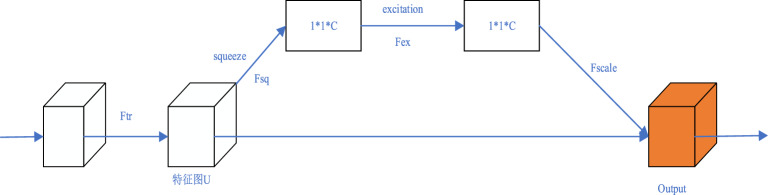
Squeeze and excitation module.

Figure 4 shows the main structure of the Squeeze and Exception (SE) module. The main operations of the SE module are squeeze and extraction, which mainly reconstruct the original features, allowing the model to directly learn channel features and obtain more important channel information. The main steps for extracting features in the SE module are as follows:

Firstly, input the feature map and generate the feature map U through $F_{{{\text{tr}}}}$ operation.

Secondly, perform the squeeze operation, which is achieved through global average pooling. Global average pooling compresses the two-dimensional features ($H \times W$) of each channel into a real number. After formula (4), the feature map is changed from ($H \times W \times C$) to ($1 \times 1 \times C$).

6 $$z_{c} = F_{sq} (u_{c} ) = \frac{1}{H \times W}\sum\limits_{i = 1}^{H} {\sum\limits_{j = 1}^{W} {u_{c} (i,j)} }$$

Next, perform an excitation operation to capture the relationship between feature channels. This step is completed through two fully connected layers $W_{1}$ and $W_{2}$. The first fully connected layer is used to reduce dimensions and is activated through ReLU, while the second fully connected layer is used to restore it to its original dimension. Obtaining channel weights s through an extraction operation finally. This operation is achieved through formula (5).

7 $$s = F_{ex} (z,W) = \sigma (g(z,W)) = \sigma (W_{2} \delta (W_{1} z))$$

Finally, the weight s obtained in the previous step is assigned to the feature map to obtain a new feature map ${\tilde{\text{x}}}_{{\text{c}}}$. After formula (6), multiply the generated weight s ($1 \times 1 \times C$) by the corresponding channel of the feature map U ($H \times W \times C$) to obtain more important feature information.

8 $$\tilde{x}_{c} = F_{scale} (u_{c,} s_{c} ) = s_{c} u_{c}$$

#### GSConv and VoV-GSCSP module

**Figure 5:**
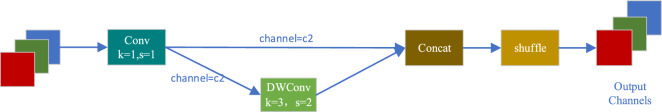
GSConv model structure.

Research has shown that most lightweight models use deep wise separable convolution (DWConv) to construct network structures. Although it reduces computational costs, the network feature extraction and fusion ability constructed by DWConv will be reduced. Li Hulin et al. proposed a new GSConv convolution method to solve this problem. Figure 5 shows the main structure of GSConv.

Firstly, perform standard convolution on the input feature map to obtain a feature map. Next, evenly divide the output channel into two halves, with half not operated and the other half undergoing deep separable convolution. Then concatenate the feature maps generated by standard convolution and DWConv convolution. Finally, channel shuffling is performed through shuffle operation to ensure information exchange.

This article proposes using the GSConv module in the Neck section, which not only reduces redundant information and computational complexity, but also improves feature extraction and fusion capabilities, improving detection accuracy.

**Figure 6:**
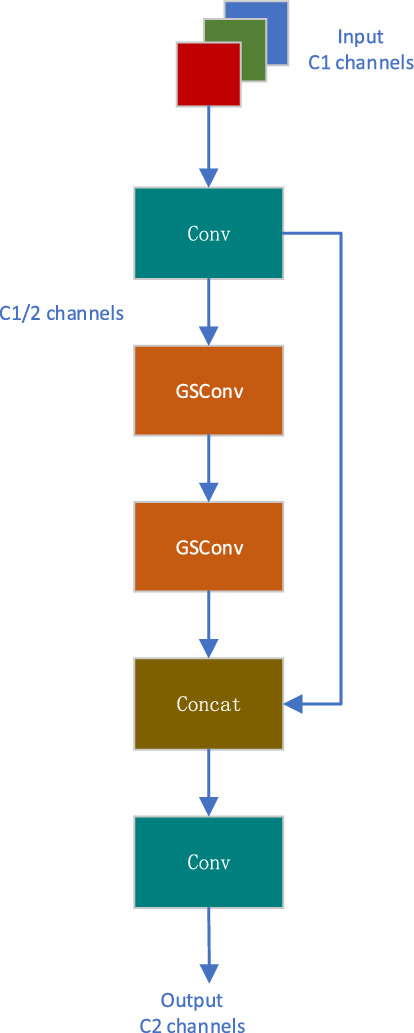
VoV-GSCSP model structure.

Li et al.^34^ proposed GSbottleneck based on GSConv. Li et al.^34^ designed the VoV-GSCSP module using a single stage aggregation method. This module reduces the complexity of computation and network structure while maintaining accuracy, making it suitable for combination with lightweight networks. The original author proposed three different VoV-GSCSP design schemes, and this article selected the VoV-GSCSP1 module with higher performance. The model structure is shown in Fig. 6.

## Results

### Experimental preparation

**Figure 7:**
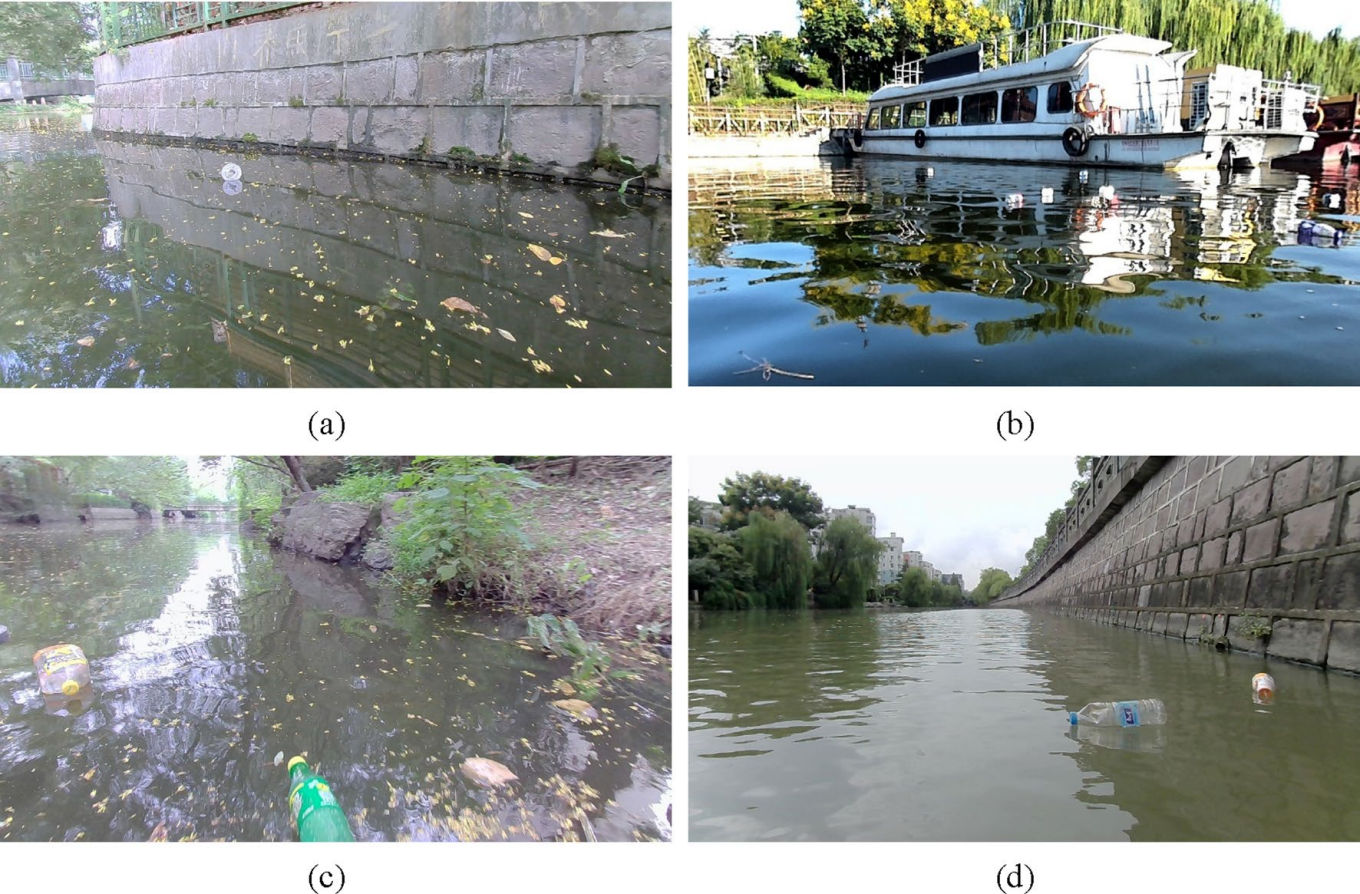
Dataset example.

Experiments in this study are based on Orca’s data set. This dataset is the world's first floating garbage dataset from the perspective of unmanned ships in real-world scenarios. This article selects the Flow-Img sub dataset, which contains 2000 images and 5271 labeled targets, meeting the requirements of this article for the dataset. Figure 7 shows an example of the Flow-Img dataset.

The experimental configuration environment for this article is NVIDIA3060, with 8 GB of graphics card memory, Windows 10 operating system, Python version 3.8, and CUDA version 10.2. The training settings are: batch size is 8, input image resolution is $640 \times 640$, The number of epoch is 400, and the training set size is 1200. Hyperparameter settings used: initial learning rate is 0.01, minimum learning rate is 0.2, learning momentum is 0.937, weight_ Decay is 0.0005, IoU training threshold is 0.5.

### Evaluating indicator

Table 1 represents a confusion matrix. By using the confusion matrix, it can be seen whether the algorithm has confused different classes. The prediction represented by TP is a positive sample, but the actual result is a positive sample, indicating that the prediction is correct. The prediction represented by TN is a negative sample, but in reality, it is a negative sample and the prediction is correct. FP indicates that the prediction is a positive sample, but the actual prediction is a negative sample, which is incorrect. The prediction represented by FN is a negative sample, but it is actually a positive sample, indicating an error in prediction. Precision and recall metrics can be derived from the confusion matrix.

**Table 1:** Prediction classification.

	Positive sample	Negative sample
Predicted positive	True Positive (TP)	False Positive (FP)
Predicted negative	False Negative (FN)	True Negative (TN)

In order to evaluate the performance of the model, this article uses six indicators to evaluate the performance of the model: Precision, Recall, Mean Precision (mAP), Parameter, Floating-point Operations (FLOPs), and Transmission Frame Rate (FPS). Among them, Parameters, Floating point Operations (FLOPs) are indicators to measure the complexity of algorithms. Parameters mainly measure the number of model parameters, while FLOPs are mainly used to measure the complexity of algorithms. Larger FLOPs indicate that algorithms have higher computational requirements and require more computational resources. The calculation formula for Precision is:

9 $$\Pr ecision = \frac{TP}{{(TP + FP)}}$$

The calculation formula for Recall rate is:

10 $${\text{Re}} call = \frac{TP}{{TP + FN}}$$

The average precision value mAP is the average of the average precision of all object classes. The calculation formula is:

11 $$mAP = \frac{{\sum\limits_{i = 1}^{N} {AP_{i} } }}{N}$$

The AP in formula (9) is the average accuracy value, which is the area enclosed by the PR curve. The calculation formula is:

12 $$AP = \int\limits_{0}^{1} {P(R)dR}$$

The calculation formula for the transmission frame rate FPS is:

13 $$FPS = \frac{1000}{{pre + ms + NMS}}$$

In the formula, pre is the image preprocessing time, including image scaling and padding, channel transformation, and dimensionality enhancement processing; Ms is the inference speed, which refers to the time from the preprocessed image input to the model output result; NMS is the post-processing time used to convert the model output results, etc.

### Result analysis

#### Experiment 1: comparison with other common models

In order to select a benchmark network, some common detection models are usually selected for comparison, and the experimental configuration is referred to Sect.  4.1. Table 2 shows the results of comparing common models with YOLOv5s.

**Table 2:** Test results of different baseline networks.

Method	P	R	mAP (ap@0.5)	Parameter	FLOPs (GB)
YOLOv3	0.814	0.789	0.824	9,301,222	23.1
YOLOv3-tiny	0.844	0.712	0.774	8,666,692	13.0
Faster RCNN	0.57	0.289	0.505	73,924,608	107.82
SSD	0.761	0.401	0.672	24,903,680	342.75
Mask RCNN	0.816	0.487	0.846	45,885,685	262.25
YOLOv8	0.816	0.77	0.812	11,125,971	28.4
YOLOv5s	0.851	0.776	0.832	7,012,822	15.8
YOLOv5m	0.834	0.773	0.821	20,852,934	47.9
YOLOv5l	0.856	0.791	0.85	46,108,278	107.6
YOLOv5n	0.826	0.774	0.815	1,760,518	4.1
Our model	0.887	0.787	0.849	878,486	1.5

Comparing the YOLOv5s algorithm with the YOLOv3 and YOLOv3-tiny algorithm models, the mAP values of the YOLOv5s algorithm increased by 0.8% and 5.8% respectively. Comparing the YOLOv5s algorithm with Faster RCNN, ssd, and YOLOv8, the mAP values of the YOLOv5s algorithm increased by 32.7%, 14%, and 2%, respectively. Comparing YOLOv5s with maskrcnn, the mAP value of Mask RCNN is better than YOLOv5s, but the accuracy and regression rate of maskrcnn are lower than YOLOv5s. The most important thing is that the parameter count of maskrcnn is 6.5 times that of YOLOv5s, which does not comply with the principle of lightweight in this article. Therefore, compared with other technologies, YOLOv5s is chosen as the basic model in this article.

Comparing YOLOv5s with YOLOv5m, YOLOv5l, and YOLOv5n, the results showed that the mAP values of YOLOv5s were 1.1% and 1.7% higher than YOLOv5m and YOLOv5n respectively. Comparing YOLOv5s with YOLOv5l, although YOLOv5l's mAP value is 1.8% higher than YOLOv5s, its parameter quantity is 6.5 times that of YOLOv5s, and its FLOPs are 6.8 times that of YOLOv5s. According to the data, YOLOv5l requires higher hardware performance and higher model complexity, which does not meet the requirements of lightweight water surface garbage detection equipment. Therefore, in order to meet the requirements of both precision and lightweight, this article chooses YOLOv5s as the basic model.

#### Experiment 2: comparative experiment on different backbone networks

To meet the requirements of lightweight, this article selects Mobilenetv3, Shufflenetv2, Ghostnet, and EfficientNet network models for comparison.

As shown in Table 3, using Ghostnet as the backbone network has higher accuracy, with mAP values higher by 4%, 1.97%, and 1.1% compared to Mobilenetv3, shuffllenetv2, and EfficientNet respectively. At the same time, its parameter count and FLOPs are also the highest. The purpose of this article is to achieve lightweight, and it is obvious that Ghostnet does not meet this requirement. So we need to choose the network structure with the lowest parameter quantity and FLOPs. According to the data in the table, using Shufflenetv2 as the network structure has the lowest number of parameters and FLOPs. The number of parameters is 0.3 times that of Mobilenetv3, 0.096 times that of Ghostnet, and 0.4 times that of EfficientNet, respectively. At the same time, its FLOPs only account for 5.2% of Mobilenetv3, 1.8% of Ghostnet, and 5% of EfficientNet. Based on the above data analysis, it is more suitable to choose Shufflenetv2 network as the backbone network.

**Table 3:** Test results of different backbone networks.

Method	P	R	mAP (ap@0.5)	Parameter	FLOPs (GB)
YOLOv5 + Mobilenetv3	0.797	0.661	0.726	1,386,812	2.5
YOLOv5 + Shufflenetv2	0.724	0.538	0.589	437,878	1.3
YOLOv5 + Ghostnet	0.82	0.707	0.766	4,531,426	7.1
YOLOv5 + EfficientNet	0.813	0.698	0.755	1,085,610	2.6

#### Experiment 3: related ablation experiment

This article selects Shufflenetv2 as the backbone network model for ablation experiments, aiming to verify the effectiveness of the improved algorithm.

In Table 4, we clearly note that the accuracy of our proposed algorithm has been improved by 3.6%, the recall rate has been improved by 1.1%, and the mAP value has also been improved by 1.7%. According to these data, the improved algorithm has higher accuracy. In addition, the parameter quantity of the improved algorithm model only accounts for 12.5% of the original data, and FLOPs only account for 9.5% of the original data, which is lighter compared to the original algorithm. In summary, the improved algorithm not only has higher accuracy, but also is more lightweight, suitable for deployment on devices with insufficient hardware resources such as unmanned ships.

**Table 4:** Ablation experiment data.

Method	P	R	mAP (ap@0.5)	Parameter	FLOPs (GB)
YOLOv5s	0.851	0.776	0.832	7,012,822	15.8
YOLOv5s + Shufflenetv2	0.724	0.538	0.589	437,878	1.3
YOLOv5s + Shufflenetv2 + SE	0.7	0.625	0.644	4,207,158	8.2
YOLOv5s + Shufflenetv2 + GSConv + VoVGSCSP	0.865	0.765	0.834	884,198	1.6
Our model	0.887	0.787	0.849	878,486	1.5

#### Experiment 4: speed comparison experiment

Table 5 shows the results of comparing the speed of the original algorithm with the improved algorithm. FPS is a parameter that describes the speed of object detection. As shown in Table 5, compared with the original algorithm, the FPS of the improved algorithm has increased by 0.043. This indicates that the improved algorithm has a faster detection speed and is more suitable for deployment on devices with high detection speed requirements.

**Table 5:** Comparison of detection speed between YOLOv5 and improved algorithms.

Method	Pre-process	ms inference	NMS	FPS
YOLOv5s	0.2	4.2	0.7	0.757
Our model	0.3	2.5	0.7	0.8

Based on the experimental data mentioned above, the detection algorithm proposed in this paper not only ensures the lightweight of the model, but also improves the detection accuracy. It is suitable for deployment on surface garbage detection equipment.

#### Experiment 5: verify the effectiveness of the improved algorithm

**Figure 8:**
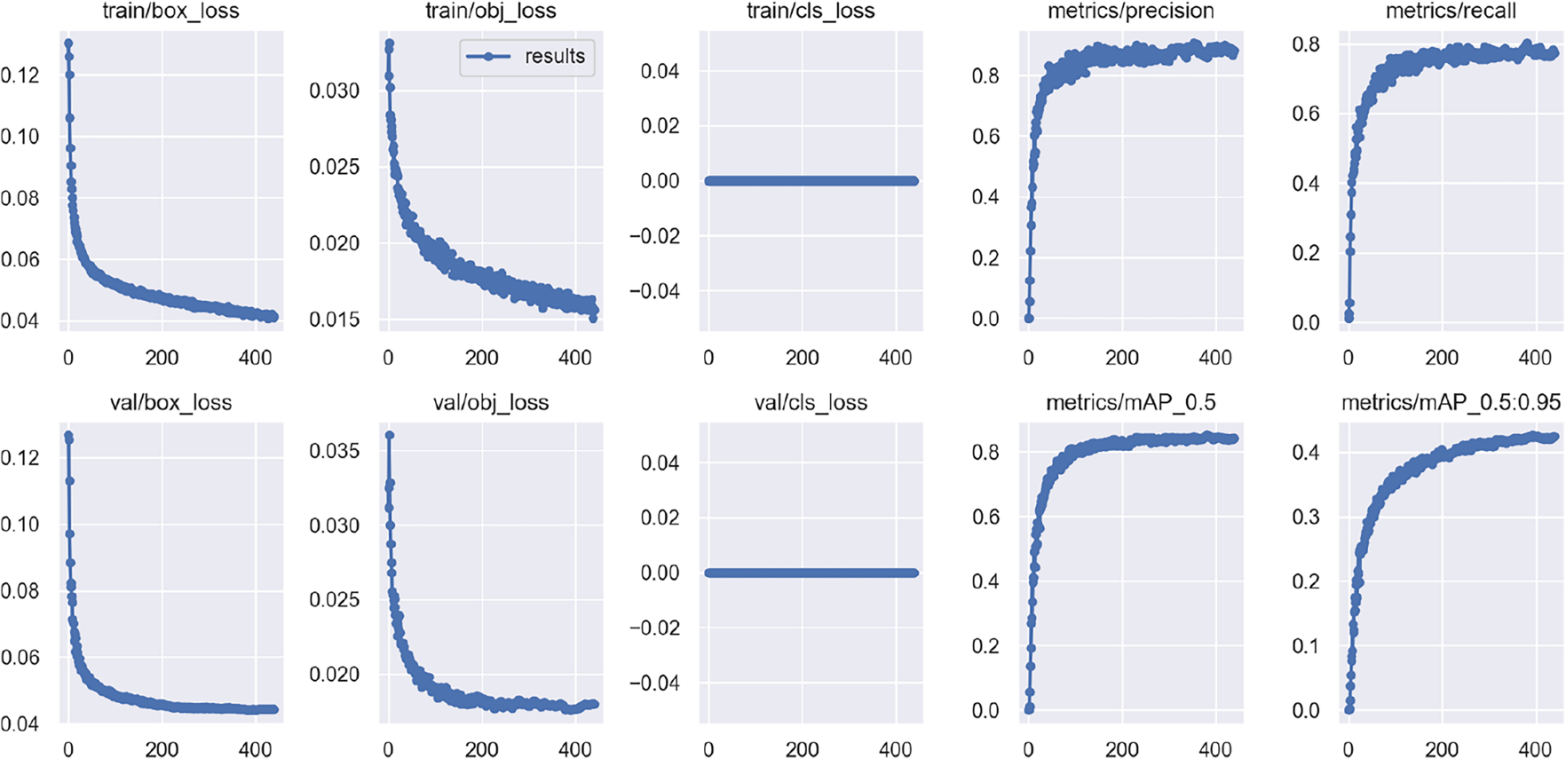
Curve diagram of the improved model training process.

Figure 8 shows the curve of the final improvement experiment training process. Train/box_ Loss, train/obj_Loss, train/class_Loss respectively represents the position coordinate prediction loss, confidence prediction loss, and category prediction loss of the training set. Val/box_Loss, val/obj_Loss, val/class_Loss respectively represents the positional coordinate prediction loss, confidence prediction loss, and category prediction loss of the validation set. From the graph, it can be seen that when the loss curve becomes stable, the P curve, R curve, and mAP curve also approach stability, indicating that the model has successfully converged at this time.

**Figure 9:**
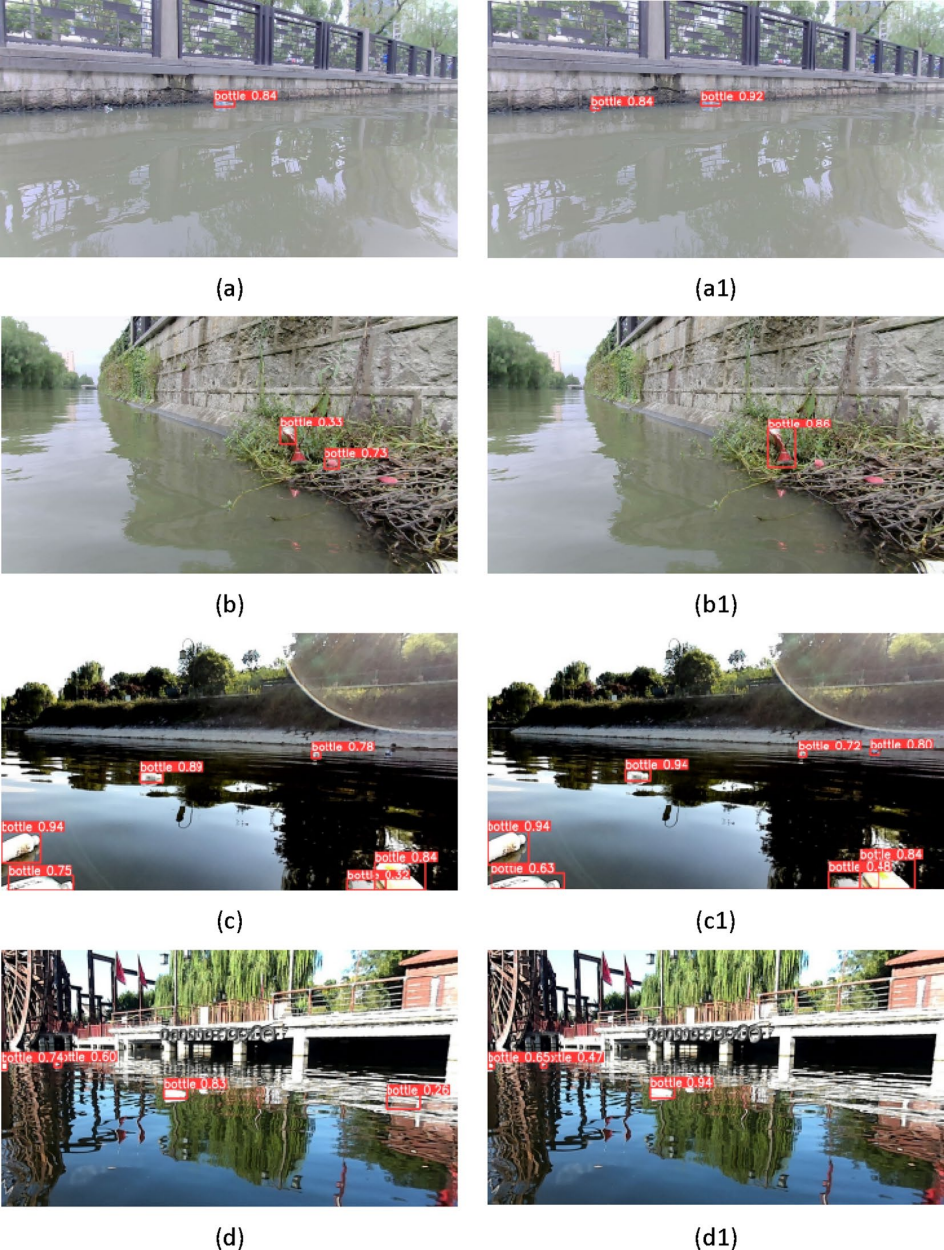
Detection result comparison (left: YOLOv5, right: improved YOLOv5).

Figure 9 shows the detection results of the YOLOv5 algorithm and the improved YOLOv5 algorithm on the test set. On the left is the detection results of the original YOLOv5 algorithm on the test set, and on the right is the improved YOLOv5 algorithm. Both algorithms can recognize large targets, but overall, the improved algorithm has higher recognition credibility, indicating that the improved algorithm proposed in this article has better recognition performance in large target detection. In the case where the detected target is far from the camera, as shown in Fig. 9a-a1, c-d1, the YOLOv5 algorithm may miss detection, but the improved YOLOv5 algorithm can still recognize targets with far distances. This indicates that the improved YOLOv5 algorithm is also better than the YOLOv5 algorithm in small target recognition. In addition, when the background contains more interfering substances, as shown in Fig. 9b-b1, the YOLOv5 algorithm may experience false positives, but the improved YOLOv5 algorithm significantly reduces its false positives.

**Figure 10:**
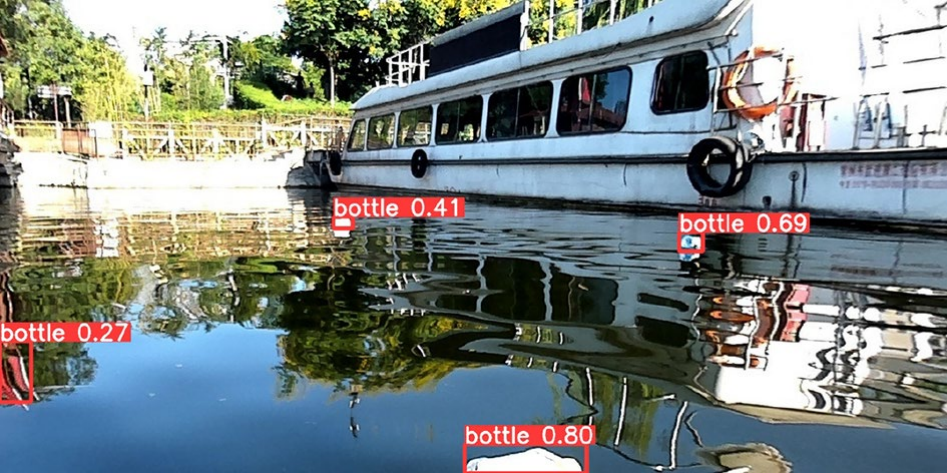
Improved algorithm detection results.

The algorithm proposed in this article maintains high detection accuracy and low false detection and missed detection rates in most cases. However, in a large number of experiments, we found that when encountering strong lighting or water reflections with dark colors, the detection performance may be reduced. As shown in Fig. 10, when the light is too strong, the plastic garbage in the distance becomes more transparent and harder to detect; when the colors of objects on the shore are too bright and reflected on the water, they may cause false detections. Although this situation occurs infrequently, it can still have a certain impact on the detection performance.

## Apply

**Figure 11:**
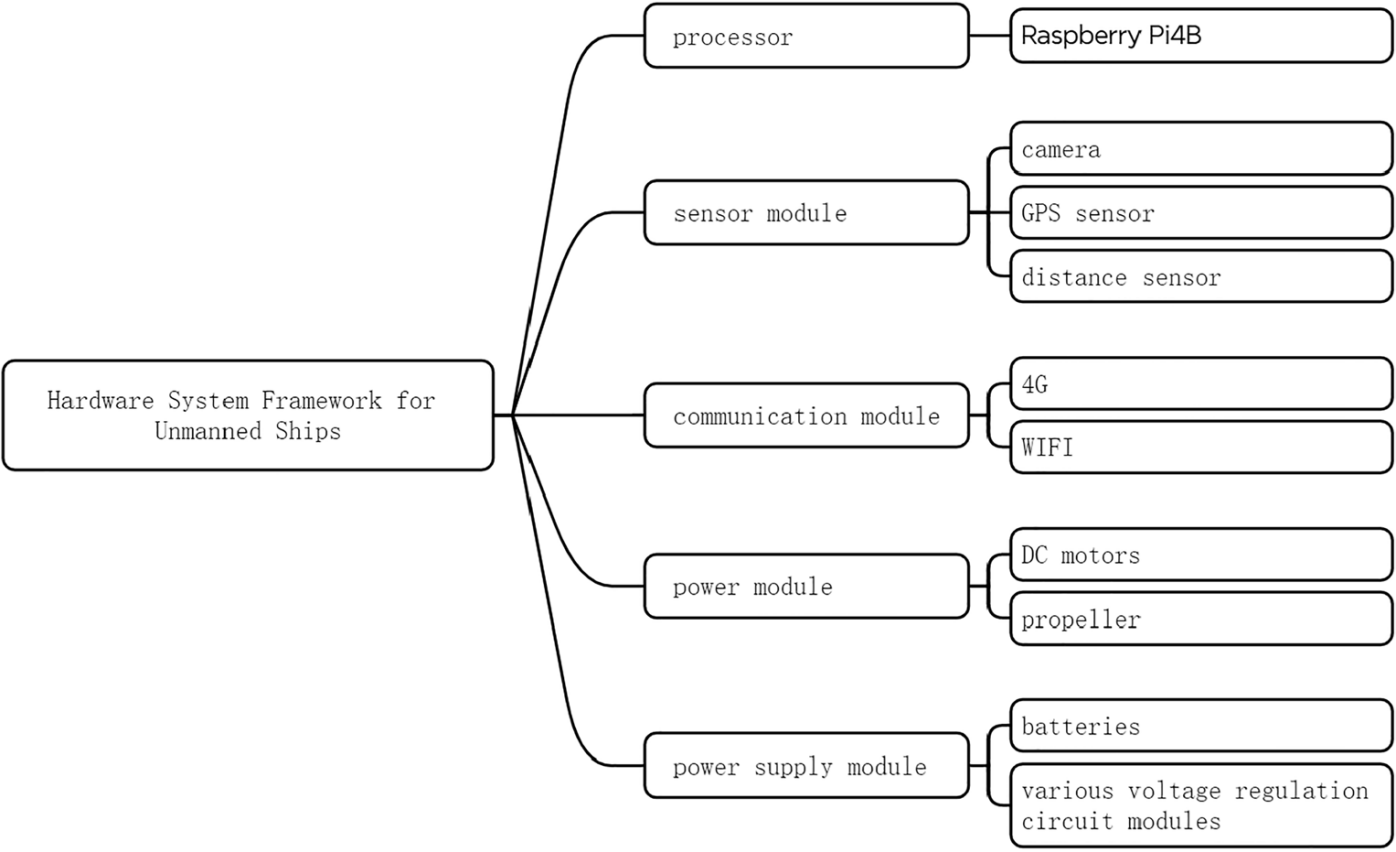
Hardware system framework for unmanned ships.

This article aims to establish a hardware platform on the unmanned ship to achieve autonomous litter detection. This article designs an unmanned ship garbage detection control system as shown in Fig. 11, which includes a processor, sensor module, communication module, power module, and power supply module.

The Raspberry Pi 4B is produced by the Broadcom Corporation and features the BCM2711 chip, which integrates a GPU and CPU. It offers powerful performance and extensive interfaces, making it a cost-effective choice for processing. The sensor module includes a camera, GPS sensor, and distance sensor. The camera is used to detect the water surface in real-time and transmit the captured images to the processor for processing. The GPS sensor is used to locate the position of the unmanned ship. The distance sensor can be used to measure the distance of objects to prevent collisions with other floating objects on the lake. The communication module of the unmanned ship can be used for communication between the unmanned ship and the ground control station or other unmanned ships. This module mainly consists of radio communication devices, data transmission equipment, etc. The power module of the unmanned ship mainly provides power to the unmanned ship, which includes DC motors and propellers. The power supply module mainly delivers stable current to the unmanned ship to ensure its operation. This module mainly includes batteries and various voltage regulation circuit modules.

In this article, the design of the unmanned ship aims to achieve garbage detection at a lower cost. Currently, deploying object detection algorithms on unmanned ships mainly faces these challenges. Firstly, traditional algorithms have high requirements for hardware equipment, which reduces economic efficiency to some extent. Secondly, these algorithms require a large amount of computing resources, leading to an increase in power consumption of unmanned ships and further affecting their endurance. However, the lightweight detection algorithm proposed in this article effectively solves these problems. It has relatively low requirements for hardware equipment, so it can be flexibly deployed on various unmanned ships, thereby improving economic efficiency. More importantly, compared with the original algorithm, the lightweight algorithm requires significantly less computing resources, which helps to improve the endurance of unmanned ships. The last challenge encountered in deploying the algorithm is that the team has just completed the hardware setup and has not yet applied it on the unmanned ship. However, the team plans to conduct tests in the future to verify the reliability of the algorithm.

## Conclusion

In order to solve the problems of slow computing speed and small storage space caused by insufficient hardware resources of unmanned ships, this paper proposes a lightweight water surface garbage detection model based on YOLOv5. Firstly, the original network structure of YOLOv5 was replaced with a Shufflenetv2 + SE network structure. Verified through experiments this model achieved lightweight, with the parameters decreased by 93%, and FLOPs only account for 9.5% of the original data. In addition, in the Neck using the GSConv module to replace the Conv module section and the VoV-GSCSP module to replace the C3 module further improved the detection accuracy, with the mAP increased by 1.7%, the speed increased by 4.3%. This indicates that the improved model not only meets the requirements of lightweight and high-precision, but also improves the detection speed, solves the problem of insufficient hardware conditions and slow detection speed of the device. Compared with the original algorithm, it is more suitable for deployment on surface garbage detection devices.

In addition, this article introduces a new perspective for future research. As the improved algorithm cannot adapt to strong lighting environments and may misdetect water reflections, in future research, we can consider preprocessing the image by reducing its brightness and then using the mean shift filtering algorithm to process the water reflections before detection.

## Data Availability

Under reasonable requirements, the dataset used in this study can be obtained from the corresponding authors.
